# Presynaptic Terminal Alterations in Concave and Convex Spinalis Muscles: A Pilot Exploratory Study in Advanced Scoliosis

**DOI:** 10.3390/jcm15124532

**Published:** 2026-06-11

**Authors:** Sebastian L. Schubert, Xiaoying Chen, Zhanyang Liang, Aline Müller, Frank Hildebrand, Miguel Pishnamaz, Mahtab Nourbakhsh

**Affiliations:** 1 Clinic for Orthopedics, Trauma, and Reconstructive Surgery, RWTH Aachen University Hospital, 52074 Aachen, Germany; seschubert@ukaachen.de (S.L.S.); fhildebrand@ukaachen.de (F.H.); mpishnamaz@ukaachen.de (M.P.); 2 Institute of Pathology, RWTH Aachen University Hospital, 52074 Aachen, Germany; xchen@ukaachen.de (X.C.); zliang@ukaachen.de (Z.L.); almueller@ukaachen.de (A.M.)

**Keywords:** adolescent idiopathic scoliosis, neuromuscular junction, presynaptic terminal, spinalis muscle, neurofilament M, synaptic vesicle glycoprotein 2

## Abstract

**Background/Objectives**: Presynaptic terminals (PTs) in the neuromuscular junction (NMJ) are essential regulators of skeletal muscle function and are responsible for the translation of electrical impulses from motor neurons into muscle contraction. The present exploratory study aimed to compare PT adaptations in spinalis muscle samples from the concave and convex regions of the spine in three cases of advanced scoliosis, which exhibited marked asymmetry in muscle development. **Methods**: Spinalis muscle sample pairs were retrieved after surgical procedures and subjected to immunofluorescence (IF)-based spatial analysis of PTs, histological assessment of muscle fibers, and expression analyses of inflammatory and neurotrophic proteins. **Results**: IF images revealed distinct differences in PT parameters between spinalis samples obtained from the corresponding concave and convex sides of spinal deformities. Advanced statistical models revealed a consistent tendency for concave spinalis muscles to develop lower PT numbers, along with decreased expression of relevant components, neurofilament M, and synaptic vesicle glycoprotein 2. Moreover, these impairments were accompanied by increased expression levels of IFN alpha, which has been previously implicated in NMJ disorders, neuropathies, and myopathies. **Conclusions**: In the concave regions of spinal deformities, continuously compressed spinalis muscles may be particularly susceptible to PT alteration and denervation. However, comprehensive multicenter validation studies are required to better define the relationships among PT alterations, IFN alpha expression, and muscle tissue compression.

## 1. Introduction

Scoliosis is an abnormal three-dimensional deformity of the spine characterized by lateral curvature and vertebral rotation, resulting in the formation of convex and concave regions in the thoracic and/or lumbar spine [1]. The convex side typically shows muscle prominence, whereas the concave side is relatively tighter because of continuous compression. The severity of the spinal deformity is primarily assessed using X-ray–based measurements of lateral curvature and vertebral rotation, known as the Cobb angle. This angle typically ranges between 10 and 50° and is classified as mild (10–25°), moderate (>25–45°), or severe (>45°) [1]. Further classifications include the Risser stage, which describes progression toward skeletal maturity, and Lenke curve types [2,3]. Management strategies for scoliosis range from conservative treatments, such as physiotherapy and brace therapy, to surgical procedures. While conservative therapies are indicated by milder deformities, lateral curves exceeding 50° are generally considered an indication for surgical intervention to prevent severe curve progression and associated secondary complications, including impaired pulmonary function. However, the prevalence of scoliosis cases with a Cobb angle greater than 40–50° is low in the general population, occurring in fewer than 0.1% of adolescents [1].

Various hypotheses have been proposed in the literature regarding the onset and pathogenesis of the most common form of pediatric scoliosis, adolescent idiopathic scoliosis (AIS) [4,5,6,7,8,9,10]. Several components of the human spine have been implicated in the initiation and progression of spinal curvature, including symmetric paraspinal muscle atrophy and fat tissue deposits (convex and concave) [4,5,6], intervertebral disk deformity [7], and genetic predisposing variants in the glycine transporter 1 gene that affect glycinergic neurotransmission [10]. Furthermore, neuromuscular impairments may also cause abnormalities in visual, vestibular, proprioceptive, and postural control asymmetry of the transverse-spinalis muscle [8,9]. Recently, electromyographic assessment of several spinal muscles in AIS patients revealed that three-dimensional spinal deformity is associated with asymmetrical activation of the paraspinal muscles [11]. Moreover, a recent systematic review provided evidence for multi-level neuromorphological alterations in the somatosensory and vestibular systems of AIS patients [12].

Neuromuscular junctions (NMJs) are specialized chemical synapses that connect motor neurons with skeletal muscle fibers, enabling rapid and reliable transmission of the neural signals required for voluntary movement and involuntary reflexes. The dynamics of signal transmission at NMJs are regulated by three functional domains: the presynaptic terminal (PT), which contains synaptic vesicles loaded with the neurotransmitter acetylcholine; the synaptic cleft, which contains extracellular matrix (ECM) components; and the postsynaptic muscle fiber membrane, which contains acetylcholine receptors (AChRs) [13]. Additionally, NMJs are surrounded by different cell types, such as macrophages, that may contribute to early damage sensing, inflammation, and tissue repair [13]. The structural integrity of the PT has been assessed in various human skeletal muscle tissues and has been demonstrated to be significantly correlated with muscle function, age, and the expression of neurotrophic factors [14,15,16,17,18]. Thus, a detailed analysis of PT structure and remodeling can serve as an indicator of skeletal muscle activity and neuromuscular performance. The current protocols for PT assessment rely on immunofluorescence-based detection of two well-established protein markers in human skeletal muscle tissue sections: neurofilament M (NF-M) and synaptic vesicle glycoprotein 2 (SV2). The resulting immunofluorescence images enable detailed image-based analyses of PT solidity, fragmentation, signal intensity, and spatial distribution [5,17,18].

NMJs are often located near the midpoint of skeletal muscle fibers; however, their density and distribution within skeletal muscle tissue strongly depend on myofiber orientation, also known as pennation [19]. This inconsistency complicates the comparative assessment of NMJs in different muscle tissues of the same individual or across cohorts. Nevertheless, strong efforts have been made to study the formation of NMJ components and their relevance for neuromuscular performance [17,18]. In particular, PT remodeling has been associated with neuromuscular deficiencies related to age or diseases such as autoimmune myasthenia gravis (MG) and congenital myasthenic syndromes (CMSs) [17,20,21].

The investigation of NMJ morphology in individuals with scoliosis is constrained by the limited availability of suitable tissue samples. A previous study examining PT formation in 15 AIS cases reported no significant differences among various types of paraspinal muscles from the convex and concave sides of the deformity [22]. Based on this, we hypothesized that the effects of spinal deformities on PT formation may be restricted to the spinalis dorsi (spinalis) muscle, a central component of the paraspinal muscle group spanning the last two thoracic and the first two lumbar vertebrae. The spinalis muscle develops early during the fetal period (4th week) as the most medial and smallest component of the erector spinae group and is composed of longitudinal muscles that are responsible for extension, flexion, rotation, and stabilization of the vertebral column. The spinalis is subdivided into three regional parts: the well-developed thoracis, often poorly developed cervicis, and capitis, which merges with the semispinalis capitis muscle. Thus, there are individual variations in the arrangement of these regions, with diverse overlapping segmentations and insertions leading to contrasting innervation patterns [23]. In the present exploratory study, we performed a comparative analysis of spinalis muscle specimens from both sides of the deformity in three advanced cases of scoliosis to determine whether PT characteristics differ between concave and convex spinalis muscle tissues.

## 2. Materials and Methods

### 2.1. Tissue Specimens

During the study, only three patients with scoliosis (Cobb angle > 50°) treated at the Clinic for Orthopedics, Trauma, and Reconstructive Surgery, RWTH Aachen University Hospital, Germany, provided informed consent and sufficient spinalis muscle tissue specimens. Relevant information on the study participants, including age, sex, Cobb angle, Lenke and French Risser deformity classification, is presented in Table 1. Each sample was placed in a sterile container and transferred to the laboratory immediately after excision. Sample collection and analyses were conducted following approval by the Medical Ethics Committee of RWTH Aachen University prior to sample collection (No. EK206/09). Written informed consent was obtained from all participants and surgeons before surgery and was archived in the Centralized Biomaterial Bank (cBMB) at RWTH Aachen University Hospital.

### 2.2. Quantification of Neurotrophic Factors and Chemokines in Tissue Extracts

According to the manufacturer’s instructions, 2.5 mg of tissue was processed to obtain tissue extracts using two preconfigured ProcartaPlex assays (EPX040-15828-901 and EPX160-12176-901; Thermo Fisher Scientific, Waltham, MA, USA). The concentrations of neurotrophic factors, ciliary neurotrophic factor (CNTF), brain-derived neurotrophic factor (BDNF), and nerve growth factor-beta (NGF beta), as well as those of inflammatory factors, eotaxin, growth-regulated oncogene (GRO) alpha, interferon (IFN) alpha, interleukin (IL)-1 alpha, IL-1RA, IL-7, IL-8, IL-15, IL-31, interferon-gamma induced protein (IP)-10, monocyte chemoattractant protein (MCP)-1, macrophage inflammatory protein (MIP)-1 alpha, MIP-1 beta, regulated on activation, normal T-cell expressed and secreted (RANTES), and stromal cell-derived factor (SDF)-1 alpha were obtained as the means of four independent multiplex immunoassay measurements. ProcartaPlex Analysis App (Thermo Fisher Scientific, USA) was used to assess bead counts, detect and correct errors, validate standard curves, and account for sample matrix effects.

### 2.3. Staining with Hematoxylin and Eosin (HE Staining)

The tissue samples were embedded in paraffin after dehydration through ascending ethanol concentrations (70%, 96%, and 100%). A SLIDE4003E microtome (pfm Medical, Cologne, Germany) was used to cut 5 µm sections from paraffin blocks. Sections were mounted onto glass slides, deparaffinized, and stained with hematoxylin for 5–10 min, rinsed in warm water for 10 min, stained with 0.3% eosin for 5 min, and washed with distilled water using an automated slide stainer (Gemini, Thermo Fisher Scientific, Waltham, MA, USA). After staining, slides were dehydrated through ascending ethanol concentrations (70%, 96%, and 100%), cleared in xylene, and covered with glass cover slips.

### 2.4. Immunofluorescence (IF) Staining

Paraffin-embedded tissue sections were deparaffinized and heated in citrate buffer (pH 6.0) for 30 min for antigen retrieval. Slides were cooled in distilled water, rinsed twice in PBS containing 0.1% Tween-20 (9127.1, Carl Roth, Karlsruhe, Germany), permeabilized in 2% Triton X-100/PBS for 15 min, and blocked using UltraCruz Blocking Reagent (Santa Cruz Biotechnology, Dallas, TX, USA) for 60 min at room temperature. Slides were incubated in diluted primary PT antibodies 2H3 (1:100; AB_531793; DSHB, Iowa City, IA, USA) and SV2 (1:100; AB_2315387; DSHB) in UltraCruz Blocking Reagent (Santa Cruz Biotechnology) overnight at 4 °C. Slides were washed and incubated in diluted secondary antibodies—anti-rabbit IgG Alexa Fluor 488 (1:100; ab150081, Abcam, Cambridge, UK) and anti-mouse IgG Alexa Fluor 594 (1:200; ab150120, Abcam) in UltraCruz Blocking Reagent for 60 min. Slides were washed again, counterstained with 0.1% DAPI (D9542, Sigma-Aldrich, St. Louis, MO, USA) in PBS for 5 min, mounted using Immu-Mount (9990402, Thermo Fisher Scientific), and sealed with glass coverslips.

### 2.5. Imaging and Image Analysis

An automated microscope (DM6000B; Leica Microsystems, Wetzlar, Germany) equipped with an integrated digital camera was used for microscopy and imaging of hematoxylin and eosin (H&E)-stained and immunofluorescence (IF) sections. A 340–380 nm filter for DAPI, a 450–490 nm filter for anti-rabbit IgG Alexa Fluor 488, and a 590 nm filter for anti-mouse IgG Alexa Fluor 594 fluorescence images were used to acquire IF images. Diskus software (version 10; Leica Microsystems) was used to process and merge images. Three to seven non-overlapping cross-sectional muscle fields (each covering 0.252 mm^2^) of each individually stained section were acquired at 20× magnification using identical exposure settings for all samples. Areas not consisting of skeletal muscle fibers were strictly excluded. Quantitative analysis of presynaptic terminals (PTs) was performed independently by two blinded investigators. Isolated single PTs were verified using transmitted light microscopy, followed by definition and marking of regions of interest (ROIs). ROIs identified by both investigators were merged for subsequent IF signal analysis. Image analysis was performed using Fiji/ImageJ (version 1.54p; National Institutes of Health, Bethesda, MD, USA; accessed 20 November 2024) to obtain morphometric and fluorescence-intensity parameters using the built-in analysis tools. Raw data were exported, processed, and summarized using Microsoft Excel 365 (version MSO; Microsoft Corporation, Redmond, WA, USA).

### 2.6. Statistical Data Analysis

All statistical analyses were performed using R (version 4.4.2; R Foundation for Statistical Computing, Vienna, Austria). Descriptive statistics were used to assess differences in myofiber characteristics between sample pairs. Repeated subsampling was performed to assess the lowest number of view fields (k = 4) per sample pair for robust PT parameters analysis. We applied different statistical methods to handle multi-level data variance, define conditional estimates, and enable comparative PT assessments. Linear mixed-effects models were adjusted at the PT level with group as a fixed effect and patient/field as nested random effects under restricted maximum likelihood (REML). IF intensity-related variables were log10-transformed to conform to the data and increase the validity of the statistical analyses. After side-wise aggregation, paired differences (convex/concave) were analyzed using the Hodges–Lehmann (HL) estimator and Wilcoxon signed-rank tests with 95% confidence intervals. Multiple testing was adjusted by the Benjamini–Hochberg method. Inflammatory and neurotrophic protein expressions were subjected to log10-transformation and z-score standardization. Paired differences were evaluated using the Hodges–Lehmann estimator and Wilcoxon signed-rank test, and associations between Δanalyte and ΔPT metrics were assessed using Spearman correlation.

## 3. Results

The density and formation of PTs in the spinalis muscle of individuals with scoliosis have not been reported before, largely because of the limited number of these patients who undergo surgical treatment. Here, we analyzed spinalis muscle tissue samples from three scoliosis patients who provided written informed consent to donate their excised tissues for experimental use. The inclusion of control donors is not feasible, as healthy spinalis muscle is not subject to surgical removal, and its collection would raise significant ethical concerns. The severity of the deformities was assessed using full-spine standing X-ray and CT imaging (Supplementary Materials, Figure S1A–C) before surgery. Only sample pairs from the concave and convex sides of the lumbar or thoracic deformities were retrieved immediately after surgery and analyzed. Table 1 provides relevant information on the study participants, including age, sex, Cobb angle, Lenke and French Risser deformity classification, and consistent tissue sample designations used in the present study. P1 and P2 samples were isolated from the lumbar end of the spinalis muscle. P3-T and P3-L represent two samples from P3, each obtained from the thoracic and lumbar ends of the spinalis, respectively.

### 3.1. Myofiber Growth in Spinalis Muscles in Patients with Scoliosis

**Figure 1:**
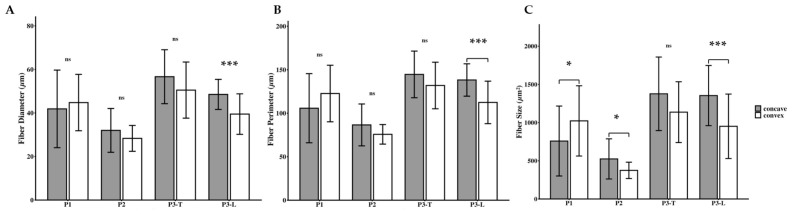
Myofiber characteristics of concave and convex spinalis muscle samples from patients with AIS. The tissue samples (x-axes) were subjected to H&E staining, and the resulting light microscopy images were analyzed to calculate the mean ± SD of myofiber diameter (**A**), perimeter (**B**), and area (**C**) in randomized view fields of 0.255 mm^2^. Nonsignificant (ns) and statistically significant differences (*p* ≤ 0.05 *; *p* ≤ 0.001 ***) between the corresponding concave (gray bars) and convex (white bars) sides are indicated. All the data sets and details of the statistical procedures are provided in the Supplementary Materials (Table S1).

The histopathological features of the spinalis muscle samples, namely, P1-convex, P1-concave, P2-convex, P2-concave, P3-T-convex, P3-T-concave, P3-TL-convex, and P3-L-concave, from three individuals with scoliosis were analyzed using H&E staining. The data and their statistical significance are provided in the Supplementary Materials (Table S1). As summarized in Figure 1A,B, we detected no significant differences between the myofiber diameters or perimeters of the corresponding convex and concave samples from patients 1 and 2 (P1 and P2, respectively). However, the mean diameter, perimeter, and area of myofibers from the lumbar end of convex spinalis tissue of patient 3 (P3) were significantly lower than those from the corresponding concave sample (diameter: *p* = 0.0002; perimeter: *p* = 0.0001; area: *p* = 0.0008). Less significant side-dependent differences in mean myofiber size were observed in P1 (*p* = 0.0325) and P2 (*p* = 0.0140). These findings suggest that the measurement of myofiber area size may provide more consistent data for detecting significant differences between concave and convex deformities of the spinalis muscles.

### 3.2. PT Analysis of the Spinalis Muscle in Patients with Scoliosis

**Figure 2:**
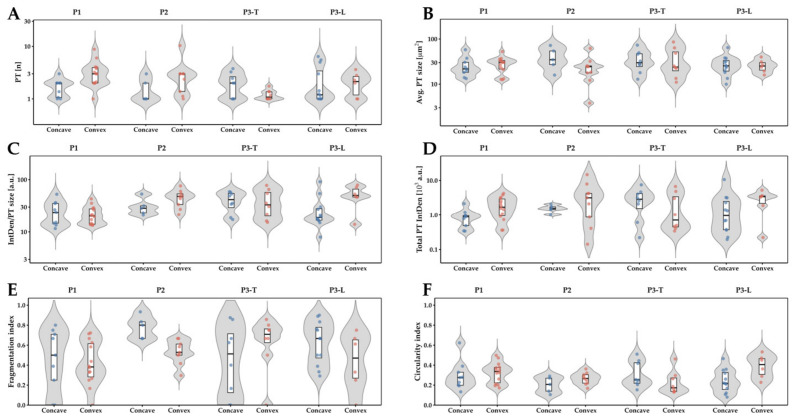
PT formation in the spinalis muscle slightly differs between the concave and convex sides of the deformity in patients with scoliosis. Concave and convex (x-axis) tissue samples—P1, P2, P3-L, and P3-T (top of the panels)—were subjected to immunofluorescence staining to obtain the medians of different PT image parameters (y-axes) per vision field (0.252 mm^2^). Violin plots illustrate the field-level distribution of each PT parameter, including the number of detectable PTs (PT, (**A**)), PT average area size (avg. PT size, (**B**)), integrated PT IF densities normalized to PT size (IntDen/PT size, (**C**)), total integrated PT IF densities (Total PT IntDen, (**D**)), PT fragmentation index (FI, (**E**)), and PT circularity index (CI, (**F**)), with summary statistics presented as medians and interquartile ranges. The blue and red dots indicate the medians of the parameters from each sample (top of diagrams) from the corresponding concave and convex regions (x-axes) of the deformities, respectively. Representative IF images are provided in the Supplementary Materials (Figure S2A,B).

For PT analysis, tissue samples were embedded in paraffin, sectioned, and subjected to IF staining using two established PT antibodies against NF-M (2H3) and SV2 (SV2) as described previously [18]. In addition, nuclear staining with DAPI was used to locate surrounding tissue cells for guidance. To capture spatially distributed PTs, we analyzed 20 randomly selected nonconsecutive stained sections, representing a cumulative tissue depth of 500 µm. Representative images are provided in the Supplementary Materials (Figure S2). In all the stained sections, multiple nonoverlapping cross-sectional muscle fields (0.252 mm^2^ each) were imaged using identical exposure settings across all the samples. To minimize bias in image acquisition and analysis, two blinded investigators independently identified individual PTs using transmitted-light microscopy, defined regions of interest (ROIs), and performed quantitative PT analysis using corresponding IF images. Different PT image variables obtained from eight spinalis samples, namely, P1-convex, P1-concave, P2-convex, P2-concave, P3-L-convex, P3-L-concave, P3-T-convex, and P3-T-concave, are shown in Figure 2A–F.

The presented variables include the following:

- The number of detectable PTs (PT; Figure 2A) and their average size (avg. PT size; Figure 2B) within multiple view fields of ~0.252 mm^2^ capture the overall PT distribution within multiple sections of each sample.
- Integrated IF densities normalized to PT size (IntDen/PT size; Figure 2C) and total integrated IF densities (Total PT IntDen; Figure 2D), showing the cumulative expression levels of NF-M and SV2.
- The fragmentation index (FI; Figure 2E) and circularity index (CI; Figure 2F) represent the spatial formation of PTs from multiple view fields of each sample.

As shown in Figure 2, some concave/convex sample pairs (P1, P2, P3-T, and P3-L) revealed distinguishable medians (concave blue and convex red dots, respectively) and interquartile ranges (IQRs; white bars with vertical lines for minimum and maximum values). For better comparability, the x-axes for identical PT image variables were scaled consistently. The key observation from Figure 2 is that PT parameters exhibited sample- and region-specific variability, with no consistent dominance of either the concave or convex side across all patients and PT characteristics. Most importantly, this asymmetry was not specific to the patient, location, or PT characteristics. Remarkably, the total PT integrated IF density (Figure 2D) and fragmentation index (Figure 2E) differed between the concave and convex sides of all the samples (P1, P2, P3-L, and P3-T). Moreover, despite differencesbetween the concave and convex sample pairs, the data density (violin plots) did not show a clear trend toward either the concave or the convex side. Thus, these findings suggest that PT formation may differ between convex and concave spinalis muscle tissues in individual cases; however, these asymmetrical adaptations are not consistently associated with either concave or convex formation of the spinalis muscle in scoliosis patients in general. Notably, our assessment revealed PT variations within a relatively large 500 µm segment of the spinalis tissue. Thus, our findings strongly support the use of spatial analysis of the spinalis muscle rather than single-tissue-section analysis for consistent assessment in patients with scoliosis.

### 3.3. Statistical Verification of the View Field Sampling Procedure and Confounding Variables

Next, we used a sampling-based sensitivity analysis (SA) to determine the lowest number of view fields per tissue section required for consistent assessment of PT image parameters. We found that at least four different view fields were sufficient to detect the described differences. In the present study, the number of evaluated view fields per sample ranged from 5 to 14, which is higher than the minimum threshold.

To account for the hierarchical structure of the data and avoid bias in the estimation of concave/convex PT trends, we used a linear mixed-effects model with restricted maximum likelihood (REML) for side-directed effects and the nested structure of the data (fields within patients). IF intensity-related variables were log10-transformed before analyses. As summarized in Table 2, we calculated negligible variance proportions attributed to different hierarchical levels, view fields (prop_field), and patient samples (prop_patient). These findings confirm that the observed variance in PT variables primarily accounts for the significant differences between concave and convex tissue samples rather than random fluctuations at the field or patient levels. The lowest variance proportions were obtained for PT size, FI, and CI (0.0000, 0.0992, and 0.0845, respectively). Integrated densities of PTs (IntDen/PT) or integrated densities normalized to PT size (IntDen/PT size) show slightly higher variance proportions between view fields (0.1969 and 0.0992, respectively) or sample pairs (0.1734 and 0.2005, respectively), implying possible background and image noise effects.

### 3.4. Nonparametric Estimation of PT Image Data for Spinalis Muscles in Patients with Scoliosis

**Figure 3:**
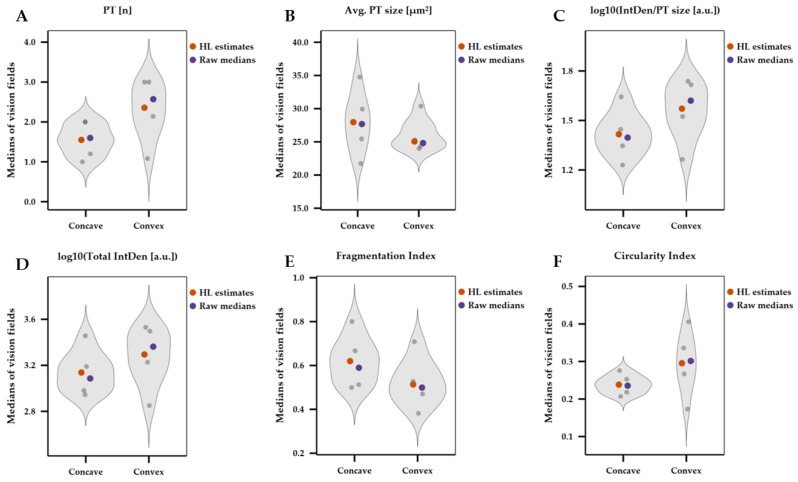
PT image variables show distinct trends toward the concave and convex spinalis muscles in patients with scoliosis. The tissue samples were subjected to immunofluorescence staining to determine the average size of the PTs per vision field (0.252 mm^2^). All data from the spinalis muscle samples were merged into two groups: concave and convex. Violin plots illustrate field-level distributions. Raw medians of vision fields (violet dots) and corresponding Hodges–Lehmann (HL) estimates (red dots) from concave and convex data sets (x-axes) are shown to distinguish observed central values from robust nonparametric estimates. Each panel shows field-level distributions, medians of vision fields, and corresponding HL estimates for the number of detectable PTs (PT, (**A**)), PT average area size (avg. PT size, (**B**)), integrated PT IF densities normalized to PT size (IntDen/PT size, (**C**)), total integrated PT IF densities (Total PT IntDen, (**D**)), PT fragmentation index (FI, (**E**)), and PT circularity index (CI, (**F**)). Exact HL estimates with 95% confidence intervals (CIs) for the paired comparisons between concave and convex samples are provided in the Supplementary Materials (Table S2).

To quantify the trends in PT parameters toward concave or convex samples, we employed the Hodges–Lehmann (HL) estimator, which has been widely used in statistical analyses of nonparametric data that are symmetrically distributed around their medians [24]. As a robust measure of central tendency, the HL estimate represents the median of all pairwise averages (also known as Walsh averages) calculated within a 95% confidence interval (CI). The raw medians calculated directly from the data measurements and the HL estimates after adjustments for the respective PT variables are summarized in Figure 3. Compared with the concave spinalis muscle, the convex spinalis muscle tended to have a greater PT number (Figure 3A), integrated PT signal density relative to PT size (Figure 3C), total integrated PT signal density (Figure 3D), and circularity index (Figure 3F). In contrast, the fragmentation index and average PT size tended to increase in the concave spinalis muscles (Figure 3B,E). However, these estimates should be interpreted as exploratory side-specific trends rather than validated evidence, given the small sample size.

### 3.5. Asymmetric Inflammatory and Neurotrophic Protein Expression in the Spinalis Muscles of Patients with Scoliosis

**Figure 4:**
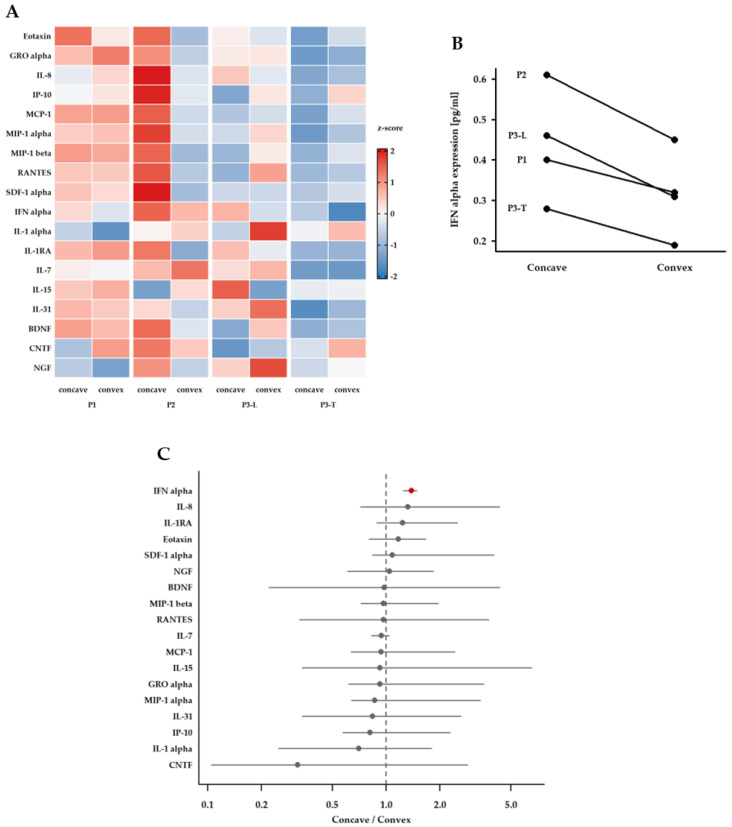
Differential protein expression patterns in the concave and convex spinalis muscles of scoliosis deformities. P1-convex, P1-concave, P2-convex, P2-concave, P3-L-convex, P3-L-concave, P3-T-convex, and P3-T-concave tissue samples were each subjected to multiplex protein assays. (**A**) Z scores normalized to the expression levels of regulatory factors after log10 transformation (y-axis) were plotted in a heatmap. (**B**) IFN alpha expression (y-axis) was higher in all the tested concave spinalis samples than in the corresponding convex samples. (**C**) Forest plot of the HL estimates for 18 different factors (y-axis). Data are presented as the mean fold difference (concave/convex ratio, x-axis) on a log10 scale. The dashed vertical line at 1.0 indicates no difference between the concave and convex samples. Fold differences with a sign stability of 1.0 are highlighted (red dots and horizontal lines), while those with lower stability are shown in gray. All the data sets and details of the statistical procedures are provided in the Supplementary Materials (Table S3).

Numerous previous studies have acknowledged the link between the expression of regulatory proteins and skeletal muscle function [25]. Our previous studies have suggested that PT formation may be affected by the levels of inflammatory and neurotrophic factors in muscle tissue [18]. Therefore, we applied a multiplex approach to assess the expression levels of various neurotrophic and inflammatory proteins in human spinalis muscle samples, which has not been previously reported. We prepared whole-tissue extracts from concave and convex samples for analysis of the expression of neurotrophic factors (CNTF, BDNF, and NGF beta) and inflammatory factors (eotaxin, GRO alpha, IFN alpha, IL-1 alpha, IL-1RA, IL-7, IL-8, IL-15, IL-31, IP-10, MCP-1, MIP-1 alpha, MIP-1 beta, RANTES, and SDF-1 alpha), as described in Section 2.2. All samples were analyzed four times independently to obtain mean concentrations. The estimated concentrations of all proteins and HL estimates with 95% CIs are provided in the Supplementary Materials (Table S3). For further analysis, we compared Z-score-normalized expression data between concave and convex sample sets in a heatmap (Figure 4A). We detected marked differences between the corresponding concave and convex samples from two samples: the lumbar deformity of patient 2 (P2) and the lumbar deformity of patient 3 (P3-L). In contrast, the expression levels of most analytes were uniform between the spinalis samples from patient 1 (P1) and the thoracic deformity samples from patient 3 (P3-T). Interestingly, IFN alpha expression was consistently higher in all the concave spinalis muscle tissue samples than in the corresponding convex samples (Figure 4B). This prompted us to estimate the overall expression trends toward the convex and concave spinalis muscles using the HL estimator as described above. The results are summarized in a forest plot, showing the two most significant differences in the expression of CNTF and IFN alpha (Figure 4C). The estimated CNTF expression was more than three times greater in the convex spinalis tissue samples. In contrast, IFN alpha expression in the concave spinalis tissue samples increased by 38.7%, with a stable sign of 1.0.

## 4. Discussion

The progression of spinal curvature in patients with scoliosis is characterized by pronounced asymmetrical activity in the paraspinal muscles, with potential involvement of NMJs [4,6,26]. The present exploratory study reports on PT formation and disposition in spinalis muscle tissues obtained exclusively from patients with concave and convex scoliosis deformities. Our study focused on comparing concave and convex muscle tissue within the same individual, thereby eliminating interindividual variability arising from cumulative biogenetic, health, and lifestyle factors. Control donors were not included, as healthy spinalis muscle is rarely removed surgically, and obtaining such samples poses significant ethical challenges. NMJs are not uniformly distributed throughout the spinalis muscle, necessitating detailed spatial tissue analysis to ensure consistent assessment of PT adaptations across multiple samples. Furthermore, the principal finding is that the concave spinalis muscle is prone to lower PT numbers and increased PT fragmentation, along with decreased expression of NF-M, SV2, and CNTF, which are involved in promoting motor neuron development [27]. These PT alterations are associated with elevated IFN alpha expression, which has previously been linked to the development or exacerbation of various neuropathy syndromes, neuromuscular junction disorders, and myopathies [28]. As our findings are based on only three cases with varying etiologies, demographics, and curve patterns, they should be interpreted cautiously and validated in larger, multicenter studies.

Several study limitations are worth noting, the foremost being the overall low number of surgical scoliosis treatments. Moreover, only a small fraction of scoliosis surgeries yield sufficient tissue samples from both sides of the deformities for detailed analysis. Therefore, robust nonparametric statistical approaches, such as the Hodges–Lehmann (HL) estimator used in the present study, are needed to mitigate the constraints associated with limited sample availability. The second limitation relates to the heterogeneous distribution of NMJs, which necessitates in-depth analysis of relatively large tissue areas, as performed in the present study. The third limitation concerns the diversity of skeletal muscle tissues from different anatomical sources. The present study provides a comparative analysis of PT formation exclusively in the spinalis muscles. However, the observed PT adaptations in the spinalis muscles may be significantly different from those in other components of the paraspinal erector spinae group. The fourth limitation concerns immunofluorescence-based PT analysis, which only implicitly translates into PT activity in the concave and convex portions of the spinalis muscle. Although this is a general restriction for all histological studies, future methodologies for local detection of action potentials in native tissues ex vivo may provide more insights into the functional adaptation of PTs in scoliosis deformities.

The effects of spinal deformities on NMJ morphology and function have rarely been studied in humans, largely because of limited access to suitable tissue samples. Only one previous study investigated NMJ morphology in paraspinal muscles—a heterogeneous group of muscles extending from the skull to the sacrum [22]. In 15 AIS cases, PT variables were compared between convex and concave paraspinal muscles without further specification of muscle type or anatomical location, and no significant differences were observed [22]. These findings support our hypothesis that PT adaptation may be specific to individual muscles and their precise location within the deformity. Consequently, stricter inclusion criteria for study specimens may increase the sensitivity for detecting differences and improve the overall significance of the data. Although such criteria may reduce sample size, the application of robust statistical models can help mitigate these limitations by uncovering underlying patterns and enabling more accurate predictions.

Previous studies revealed significant alignments between PT formation, NMJ function, and muscle activity that may potentially apply to our findings in the spinalis muscle of scoliosis cases. Endurance training was found to increase PT transmission and stability in the muscle biopsies of young athletes [29]. Similarly, sustained high-intensity exercise was shown to decrease PT fragmentation and promote PT expansion and the efficiency of neurotransmission in rodents [30]. Increased PT fragmentation and impaired expression of NF-M and SV2 have been associated with impaired muscle function owing to disease, aging, or injury [17,31]. Previously, we demonstrated that specific variables in PT immunofluorescence (IF) images were associated with impaired skeletal muscle function owing to age or obesity [18]. Our present findings indicate that concave spinalis muscle tissue tends to decrease PT density, lower NF-M and SV2 expression, and increase PT fragmentation. In accordance with the findings of previous studies, our findings suggest impaired PT activity and neurotransmission in the concave spinalis muscle in scoliosis deformities. However, it remains unclear whether impaired PT activity and neurotransmission contribute to the progression of the deformity, or whether deformity-induced compression of muscle tissue on the concave side leads to impaired PT activity.

The broad detection of protein markers in spinalis specimens (Figure 4A) revealed distinct expression patterns that may be associated with the severity of scoliosis progression. For example, P2, an 11-year-old patient with neurogenic scoliosis, exhibited rapid development of severe deformity with a Cobb angle of 70°. In the concave spinalis muscle of P2, nearly all inflammatory markers are upregulated, indicating a persistent inflammatory tissue microenvironment in the compressed muscle. In contrast, P1, a 34-year-old AIS patient with slowly progressing deformities, had low bilateral expression levels of inflammatory proteins. Moreover, P3 is a 14-year-old AIS patient who developed a primary lumbar deformity with a Cobb angle of 66° and a secondary compensatory thoracic deformity. Notably, the overall expression of inflammatory markers is greater in the spinalis muscle at the site of the primary lumbar deformity. These observations suggest that more severe and rapidly progressing scoliosis deformities may be associated with increased local muscle inflammation. However, additional cases are needed to confirm this hypothesis.

A key finding of the present study is the observed overexpression of IFN alpha in the compressed, concave spinalis muscles across all three patients; however, this finding should be interpreted with caution and requires further validation. Notably, over decades, the therapeutic use of IFN alpha has been increasingly implicated in the development of autoimmune-mediated diseases of the neuromuscular system [28,32]. Moreover, elevated IFN alpha expression was detected in muscle biopsies of juvenile dermatomyositis patients with advanced myopathies [33]. However, the mechanisms underlying IFN alpha activity in autoimmunity are not known and may strongly depend on different genetic susceptibilities yet to be identified. Nevertheless, the observed elevation of IFN alpha expression in compressed concave spinalis muscles may indicate a possible association with the PT alterations reported in this study; however, this observation requires further validation in larger multicenter studies.

## 5. Conclusions

Our study provides several findings suggesting a potential correlation between spinal deformities and PT alterations in the spinalis muscle. However, these correlations require further validation in larger and more diverse cohorts, as well as through functional analyses of PTs rather than solely structural assessments. These observations substantiate previous research, demonstrating that the pathogenesis of scoliosis is associated with neuromuscular deficiencies [4,6,26]. Similarly, the pathogenesis of neuromuscular disorders often involves NMJ deficiency and the later development of secondary scoliosis [34]. Future studies may contribute to a better understanding of the cellular origin of IFN alpha expression in spinalis muscle tissue and may offer initial insights into mechanisms underlying a potential association between IFN alpha expression and PT alterations.

## Figures and Tables

**Table 1:** Patient characteristics and tissue samples.

Patient	Sex	Age [Years]	Deformity	Cobb Angle	Sample
P1	M	34	Lenke 3CN	thoracic 76°	P1-concave/convex
			Risser 5	lumbar 66°	
P2	M	11	Lenke 1B	lumbar 70°	P2-concave/convex
			Risser 4		
P3	F	14	Lenke 1CN	thoracic 73°	P3-T-concave/convex
			Risser 4	lumbar 66°	P3-L-concave/convex

**Table 2:** Descriptive variance proportions attributed to the selected view fields (prop_field) or patient sample pairs (prop_patients).

Variable	Transformation	Prop_Field	Prop_Patient
PT size	none	0.0000	0.0000
IntDen/PT	log10	0.0006	0.1734
IntDen/PT size	log10	0.1969	0.2005
Fragmentation index	none	0.0992	0.0244
Circularity index	none	0.0845	0.0001

## Data Availability

The original contributions presented in this study are included in the article/Supplementary Materials. Further inquiries can be directed to the corresponding author.

## Supplementary Materials

The following supporting information can be downloaded at: https://www.mdpi.com/article/10.3390/jcm15124532/s1, Figure S1: Presurgical images of AIS cases; Figure S2A,B: Representative immunofluorescence (IF) images; Table S1: Myofiber characteristics in concave and convex spinalis muscle samples from scoliosis cases; Table S2: HL estimates and 95% CI of PT variables in concave and convex spinalis muscles; Table S3: Expression of different proteins in concave and convex spinalis muscles of scoliosis deformities.

## Abbreviations

The following abbreviations are used in this manuscript:

AChR	Acetylcholine receptor
AIS	Adolescent Idiopathic Scoliosis
Avg.	Average
BDNF	Brain-derived neurotrophic factor
CI	Circularity index
CMS	Congenital myasthenic syndrome
CNTF	Ciliary neurotrophic factor
ECM	Extracellular matrix
FI	Fragmentation index
GRO	Growth-regulated oncogene
IFN	Interferon
IL	Interleukin
IP	Interferon-gamma-induced protein
MCP	Monocyte chemoattractant protein
MG	Autoimmune myasthenia gravis (MG) congenital myasthenic syndromes
MIP	Macrophage inflammatory protein
NF-M	Neurofilament M
NGF	Nerve-growth factor
NMJ	Neuromuscular junction
PT	Presynaptic terminal
RANTES	Regulated on activation, normal T-cell expressed and secreted
SDF	Stromal-cell-derived factor
SV2	Synaptic vesicle glycoprotein 2
