# Supplementary material for: Presynaptic Terminal Alterations in Concave and Convex Spinalis Muscles: A Pilot Exploratory Study in Advanced Scoliosis
Source: J Clin Med. 2026 Jun 11;15(12):4532. doi: 10.3390/jcm15124532 (PMC13300889; doi:10.3390/jcm15124532)
Supplement: Supplementary file 1 [file jcm-15-04532-s001.zip › jcm-4289161-supplementary.pdf]

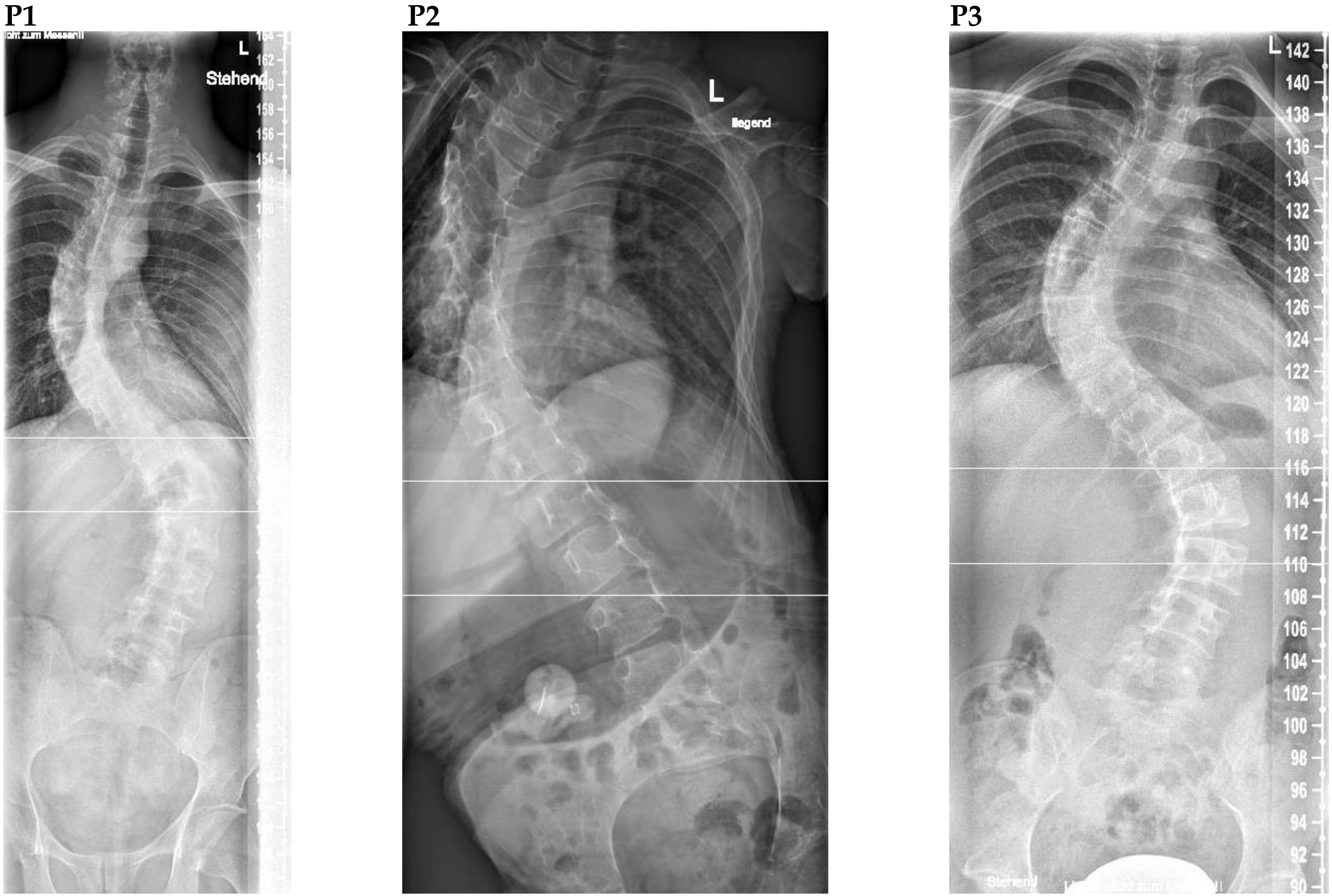

Figure S1. Presurgical full-spine standing X-ray images of three scoliosis cases (P1, P2, and P3). Each full-spine panel consists of three stitched images, as indicated by the white lines.

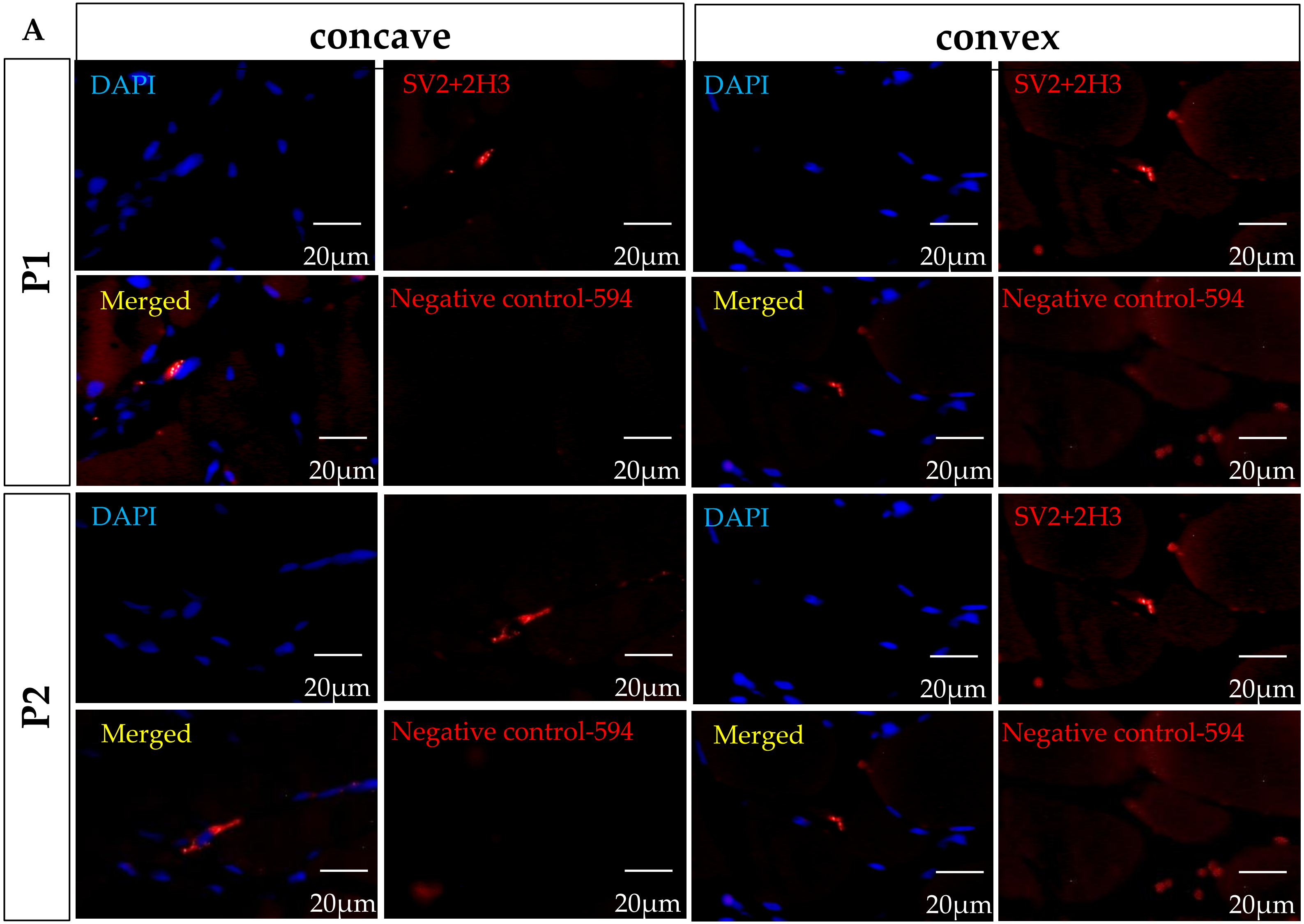

Figure S2A. Representative immunofluorescence (IF) images of postsurgical spinalis muscle tissue samples. P1 and P2 (left to the panels) from concave and convex (top of the panels) side of deformities. The tissue sections were subjected to immunofluorescence staining for neurofilament 2H3 and synaptic vesicle protein SV2 (red) together with DAPI (blue). Fluor 594-conjugated secondary antibody (red) were used to detect PTs. Staining without antibodies against 2H3 and SV2 (negative control-594) served as negative controls. Images of the same field were obtained using corresponding filters and then merged to facilitate clearer recognition of labeled structures. The white scale bars (lower right) indicate 20 μm.

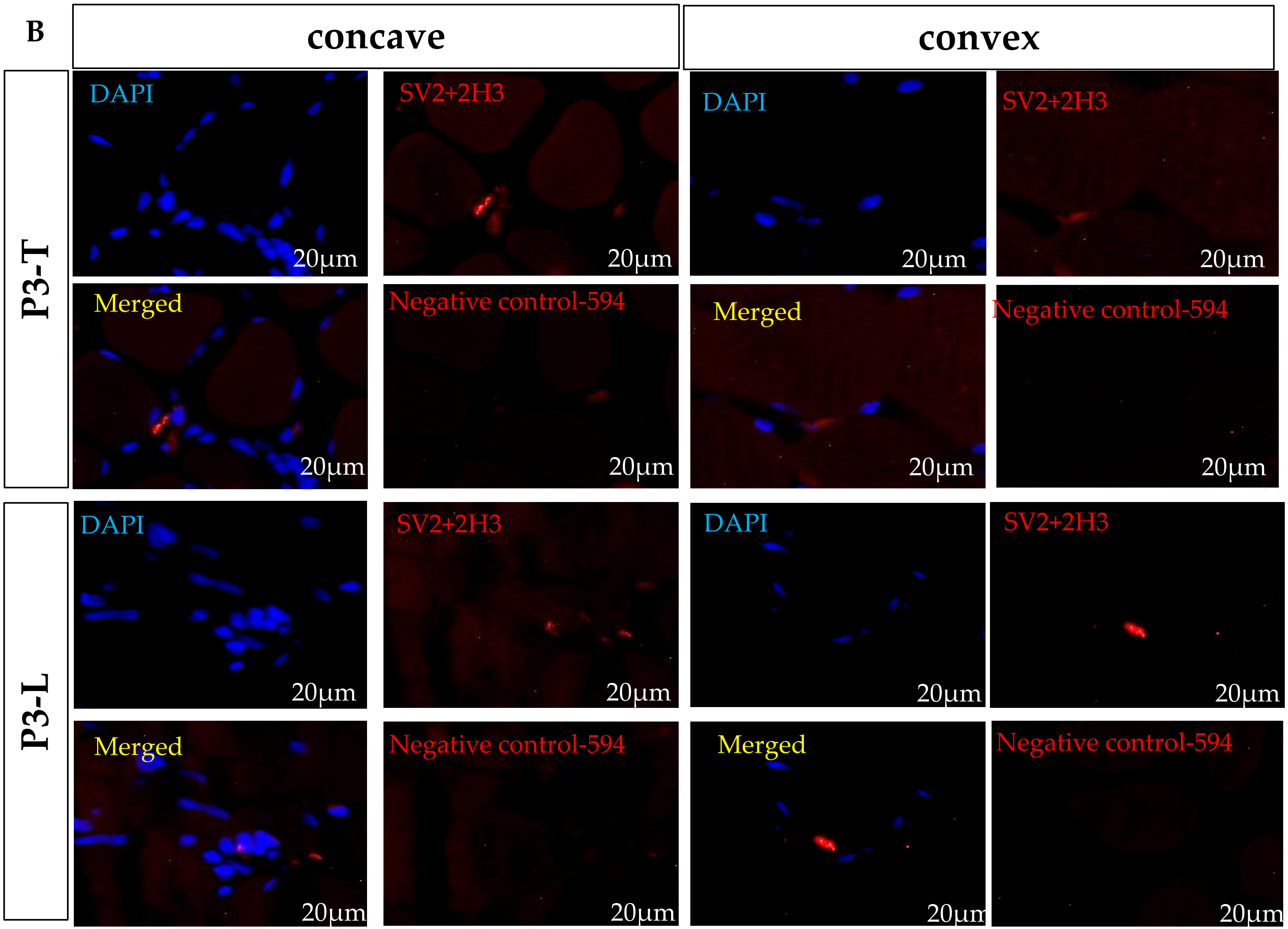

Figure S2B. Representative immunofluorescence (IF) images of postsurgical spinalis muscle tissue samples. P3-T and P3-L (left to the panels) from concave and convex (top of the panels) side of deformities. The tissue sections were subjected to immunofluorescence staining for neurofilament 2H3 and synaptic vesicle protein SV2 (red) together with DAPI (blue). Fluor 594-conjugated secondary antibody (red) were used to detect PTs. Staining without antibodies against 2H3 and SV2 (negative control-594) served as negative controls. Images of the same field were obtained using corresponding filters and then merged to facilitate clearer recognition of labeled structures. The white scale bars (lower right) indicate 20 μm.

Table S1. Myofiber characteristics in concave and convex spinals muscle samples from scoliosis cases. Tissue samples (sample) were subjected to H&E staining and resulting light microscopic images were analyzed to calculate mean ± SD of myofiber perimeters. diameters and area sizes in randomized view fields of 0.255 μm^2. Not significant (ns) and statistically significant differences ($p \leq 0.001$ ***) between corresponding concave and convex are indicated.

	**Sample**	**Concave（Mean±SD）**	**Convex（Mean±SD）**	**P-value（Welch's t-test）**
**Fiber Perimeter [μm]**	P1	105.76±39.75	122.64 ± 32.52	0.0718 (ns)
	P2	86.53 ± 24.09	75.72 ± 11.26	0.05001 (ns)
	P3-T	144.60 ± 26.82	131.86 ± 26.78	0.1065 (ns)
	P3-L	138.20 ± 18.62	112.41 ± 24.43	0.0001 (***)
**Fiber Diameter [μm]**	P1	41.87 ±17.82	44.77 ±12.94	0.4578 (ns)
	P2	31.99 ±10.06	28.32 ±5.94	0.1148 (ns)
	P3-T	56.65 ±12.39	50.49 ±12.89	0.0985 (ns)
	P3-L	48.52 ±6.91	39.48 ±9.30	0.0002 (***)
**Fiber Size [μm^2]**	P1	758.48 ±457.74	1022.25 ±459.97	0.0325 (*)
	P2	524.34 ±262.78	374.45 ±107.05	0.0140 (***)
	P3-T	1376.78 ±481.45	1136.88 ±397.95	0.0665 (ns)
	P3-L	1353.97 ±394.01	951.56 ±421.85	0.0008 (***)

Table S2. HL estimates and 95% CI of PT variables in concave and convex spinalis muscles. Side-wise PT variables were first grouped within each paired sample unit. Δ was defined as convex − concave PT variables, and Hodges–Lehmann estimates were calculated from these paired unit-level differences. HL estimates are presented as a robust effect size of paired side differences (HL(Δ), convex − concave) with 95% CI.

Variables	HL(Δ)	95%CI
**PT number**	0.957	[-0.918, 2.000]
**Ave. PT size**	-1.958	[-10.412, 8.662]
**log10(IntDen/PT size)**	1.319	[0.794, 2.652]
**log10(Total IntDen)**	1.939	[0.248, 3.538]
**FI**	-0.138	[−0.272, 0.196]
**CI**	0.060	[−0.080, 0.188]

Table S3. Expression of different proteins in concave and convex spinalis muscle of scoliosis deformities. Tissue samples P1-convex, P1-concave, P2-convex, P2-concave, P3-L-convex, P3-L-concave, P3-T-convex, P3-T-concave were each subjected to multiplex protein assays. The detected level of all proteins were normalized to 1 mg of tissue extract. The concentrations of differentially expressed proteins in concave and convex samples are presented as pg/mg. Hodges–Lehmann (HL) estimate is presented as a robust effect size of paired side differences (HL(Δ), convex − concave) with 95% confidence intervals (95% CI).

	**P1**		**P2**		**P3-T**		**P3-L**			
	**convex**	**concave**	**convex**	**concave**	**convex**	**concave**	**convex**	**concave**	**HL(Δ)**	**95% CI**
**Eotaxin (CCL11)**	1.600	2.080	1.270	2.120	1.410	1.130	1.450	1.580	0.0694	[-0.0960, 0.2224]
**GRO alpha (CXCL1)**	43.630	26.920	10.860	38.320	7.280	5.850	19.880	19.230	-0.0346	[-0.2097, 0.5475]
**IFN alpha**	0.320	0.400	0.450	0.610	0.190	0.280	0.310	0.460	0.1422	[0.0970, 0.1713]
**IL-1 alpha**	0.050	0.090	0.160	0.130	0.180	0.120	0.360	0.090	-0.1533	[-0.6020, 0.2552]
**IL-1RA**	494.010	438.610	222.370	559.570	233.940	233.580	323.380	430.340	0.0929	[-0.0516, 0.4007]
**IL-7**	1.690	1.760	2.450	2.030	1.140	1.160	2.050	1.840	-0.0259	[-0.0816, 0.0176]
**IL-8 (CXCL8)**	3.920	2.820	2.620	11.370	1.820	1.410	2.730	4.340	0.1233	[-0.1430, 0.6374]
**IL-15**	3.510	2.980	2.620	0.890	2.010	1.910	0.890	5.830	-0.0344	[-0.4688, 0.8162]
**IL-31**	3.010	3.710	0.950	2.500	0.590	0.270	8.300	2.810	-0.0747	[-0.4703, 0.4201]
**IP-10 (CXCL10)**	8.910	8.160	7.570	17.290	9.450	5.410	8.860	5.080	-0.0890	[-0.2422, 0.3586]
**MCP-1 (CCL2)**	51.800	50.190	27.770	67.490	28.720	18.260	28.310	24.010	-0.0282	[-0.1966, 0.3856]
**MIP-1 alpha (CCL3)**	0.940	0.880	0.550	1.860	0.440	0.300	0.830	0.530	-0.0631	[-0.1947, 0.5291]
**MIP-1 beta (CCL4)**	71.230	73.690	43.510	85.370	52.110	41.750	58.870	42.630	-0.0130	[-0.1401, 0.2927]
**RANTES (CCL5)**	186.540	188.400	93.810	353.660	93.840	80.240	234.500	76.560	-0.0138	[-0.4861, 0.5763]
**SDF-1 alpha**	266.950	297.260	155.060	625.480	196.760	165.700	187.590	187.580	0.0350	[-0.0746, 0.6057]
**BDNF**	16.884	21.330	7.640	33.126	4.799	3.592	15.050	3.324	-0.0108	[-0.6558, 0.6370]
**CNTF**	34.435	3.616	19.122	54.941	25.892	6.533	4.130	1.432	-0.4946	[-0.9787, 0.4583]
**NGF**	0.037	0.047	0.049	0.090	0.061	0.051	0.116	0.070	0.0198	[-0.2159, 0.2652]
